# Complete mitochondrial genome of *Conocephalus melaenus* (Orthoptera: Tettigoniidae): a tRNA-like sequence on the J-strand

**DOI:** 10.1080/23802359.2018.1424582

**Published:** 2018-01-10

**Authors:** Fei Liu

**Affiliations:** College of Life Sciences and Food Engineering, Shaanxi Xueqian Normal University, Xi’an, R.P. China

**Keywords:** *Conocephalus melaenus*, mitogenome, tRNA-like sequence, phylogeny

## Abstract

In this study, the complete mitogenome sequence of *Conocephalus melaenus* (Orthoptera: Tettigoniidae) has been decoded for the first time by next generation sequencing method. The overall base composition of *C. melaenus* mitogenome are 37.1% for A, 17.3% for C, 11.1% for G and 34.4% for T, and has high AT content of 71.5%. The complete mitochondrial genome of *C. melaenus* is 15,923 bp in length, and consisted of 13 protein-coding genes (PCGs), 22 tRNA genes, two rRNA genes and one A + T-rich region. An unusual feature of the *C. melaenus* mitogenome is the presence of a *tRNA^Ser(UCN)^*-like sequence on the J-strand, and the *tRNA^Ser(UCN)^*-like sequence have proper folding structures and anticodons sequences. Using the 13 PCGs and 2 rRNA of *C. melaenus*, together with 17 other close-related and two outgroup species, we constructed Bayesian inference phylogenetic tree to verify the accuracy and utility of new determined mitogenome sequences. The complete mitogenome of *C. melaenus* provides valuable molecular data for further phylogenetic and evolutionary analysis in Tettigoniidae.

*Conocephalus melaenus* belongs to Conocephalinae, Tettigoniidae, Orthoptera. The specimen of *C. melaenus* was collected from Xinyang, Henan, China (N 31°48′, E 114°04′) in 9^th^ August 2009, and was now deposited in Molecular and Evolutionary Lab in Shaanxi Normal University. The total DNA was isolated from the leg muscle of the specimen using the phenol-chloroform extraction method (Zhou et al. 2010). We sequenced the genome of *C. melaenus* using the next-generation sequencing technology, the raw reads and bases generated from HiSeq 2500 (Illumina, San Diego, CA) were 11,073,675 and 2.79Gb separately, and the length of one read is about 125 bp. Then the CLC Genomics Workbench 9.0 (CLC Bio, Aarhus, Denmark) was used to remove low-quality reads. Using the *Conocephalus maculatus* mitochondrial genome as the reference sequence, we assembled the mitochondrial genome of *C. melaenus* using Mira 4.0.2 and MITObim 1.7 (Hahn et al. 2013). The annotation of protein-coding genes (PCGs), rRNA genes and A + T-rich region were conducted using Geneious 9.1.2 (Biomatters Ltd., Auckland, New Zealand). tRNA genes were predicted using online software tRNAScan-SE 1.21 and MITOS (Lowe and Eddy 1997; Bernt et al. 2013).

The complete mitogenome of *C. melaenus* is 15,923 bp in length and has been deposited in GenBank (accession no. KY407794). The overall base composition of *C. melaenus* mitogenome are 37.1% for A, 17.3% for C, 11.1% for G and 34.4% for T, and has high AT content of 71.5%. It comprises 13 PCGs, 22 tRNA genes, two rRNA genes and one A + T-rich region. The orientation and gene order of the *C. melaenus* mitogenome are identical to the hypothesized ancestral arthropod and the reference *C. maculatus*. One *tRNA^Ser(UCN)^*-like sequence on the J-strand was found in the *C. melaenus* mitogenome. The *tRNA^Ser(UCN)^*-like sequence is 68 bp in length and located between *tRNA^Ser(UCN)^* and *nad1*. The *tRNA^Ser(UCN)^*-like sequence has 94.1% identity, a similar folding structure and the same anticodon sequence with *tRNA^Ser(UCN)^*. Additionally, a 356 bp intergenic spacer was found between *nad1* and *tRNA^Leu(CUN)^*. *16S rRNA* and *12S rRNA* genes are 1315 bp and 837 bp in length separately, and the control region is as long as 654 bp, with an AT bias of 79.1%.

To furthermore validate the new determined sequences and annotations, the concatenated mitogenome dataset (PCGs and rRNAs) of *C. melaenus* in this study, and together with other 19 species from GenBank, including 17 species from Tettigoniidae and two Caeliferan outgroup species were used to perform phylogenetic analysis (Figure 1). The phylogenetic tree showed that *C. melaenus* and *C. maculatus* were grouped together with a 100% bootstrap support. The complete mitogenome of *C. melaenus* provides valuable molecular data for further phylogenetic and evolutionary analysis in Tettigoniidae.

**Figure 1.**
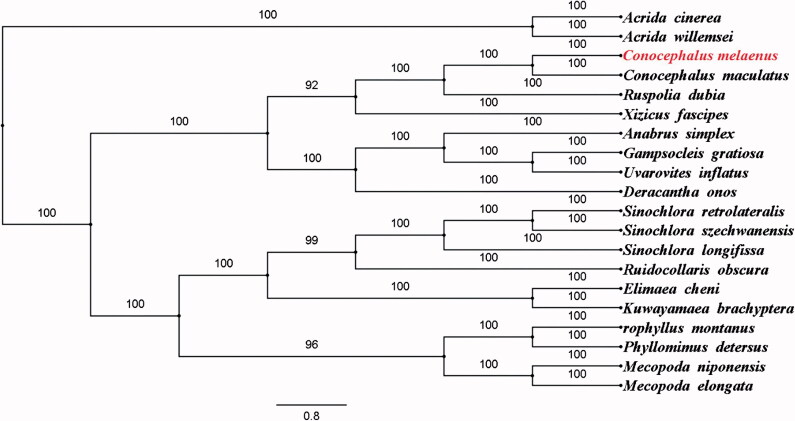
The Bayesian inference phylogenetic tree of the *Conocephalus melaenus* in this study and other 19 species including 17 from Tettigoniidae and two outgroup species based on mitochondrial PCGs and rRNAs concatenated dataset. The GenBank accession numbers for tree construction is listed as follows: *Acrida cinerea* (GU344100), *Acrida willemsei* (EU938372), *Anabrus simplex* (NC_009967), *Conocephalus maculatus* (NC_016696), *Deracantha onos* (NC_011813), *Elimaea cheni* (NC_014289), *Gampsocleis gratiosa* (NC_011200), *Kuwayamaea brachyptera* (NC_028159), *Mecopoda elongata* (NC_021380), *Mecopoda niponensis* (NC_021379), *Orophyllus montanus* (KT345951), *Phyllomimus detersus* (NC_028158), *Ruidocollaris obscura* (NC_028160), *Ruspolia dubia* (NC_009876), *Sinochlora longifissa* (NC_021424), *Sinochlora retrolateralis* (KC467056), *Sinochlora szechwanensis* (KX354724), *Uvarovites inflatus* (NC_026231) and *Xizicus fascipes* (NC_018765).

## References

[CIT0001] Bernt M, Donath A, Jühling F, Externbrink F, Florentz C, Fritzsch G, Pütz J, Middendorf M, Stadler PF. 2013 MITOS: Improved de novo metazoan mitochondrial genome annotation. Mol Phylogenet Evol. 69:313–319. 22982435 10.1016/j.ympev.2012.08.023

[CIT0002] Hahn C, Bachmann L, Chevreux B. 2013 Reconstructing mitochondrial genomes directly from genomic next-generation sequencing reads–a baiting and iterative mapping approach. Nucleic Acids Res. 41:e129. 23661685 10.1093/nar/gkt371 PMC3711436

[CIT0003] Lowe T, Eddy S. 1997 tRNAscan-SE: a program for improved detection of transfer RNA genes in genomic sequence. Nucleic Acids Res. 25:955–964. 9023104 10.1093/nar/25.5.955 PMC146525

[CIT0004] Zhou Z, Ye H, Huang Y, Shi F. 2010 The phylogeny of Orthoptera inferred from mtDNA and description of *Elimaea cheni* (Tettigoniidae: Phaneropterinae) mitogenome. J Genet Genomics. 37:315–324. 20513632 10.1016/S1673-8527(09)60049-7

